# Differential effects of arsenite and arsenate on rice (*Oryza sativa*) plants differing in glutathione S-transferase gene expression

**DOI:** 10.1007/s11356-023-28833-x

**Published:** 2023-07-24

**Authors:** Ambika Pandey, Lin-Bo Wu, Varunseelan Murugaiyan, Gabriel Schaaf, Jauhar Ali, Michael Frei

**Affiliations:** 1 Department of Agronomy and Crop Physiology, Institute for Agronomy and Plant Breeding I, Justus Liebig University Giessen, 35390 Giessen, Germany; 2 Rice Breeding Platform, International Rice Research Institute (IRRI), Los Baños, 4031 Laguna, Philippines; 3 Institute of Crop Sciences and Resource Conservation (INRES), Rheinische Friedrich-Wilhelms-University Bonn, 53115 Bonn, Germany

**Keywords:** Abiotic stress, Arsenate, Arsenite, Glutathione S-transferase, Metalloid toxicity, Reactive oxygen species, Cereals

## Abstract

Contamination of paddy soils with arsenic (As) can cause phytotoxicity in rice and increase the accumulation of arsenic in grains. The uptake and accumulation of As in rice depends on the different As species present in the soil. Plants detoxify As by conjugating and sequestering xenobiotic compounds into vacuoles using various enzymes. However, the severity of damage induced by arsenite (As(III)) and arsenate (As(V)), as well as the roles of glutathione S-transferase in detoxifying these As species in rice, are not fully understood. In this study, we developed plant materials overexpressing a glutathione S-transferase gene *OsGSTU40* under the control of the maize *UBIL* promoter. Through systematic investigations of both wild-type Nipponbare (*Oryza sativa* L., ssp. japonica) and *OsGSTU40* overexpression lines under chronic or acute stress of As, we aimed to understand the toxic effects of both As(III) and As(V) on rice plants at the vegetative growth stage. We hypothesized that (i) As(III) and As(V) have different toxic effects on rice plants and (ii) *OsGSTU40* played positive roles in As toxicity tolerance. Our results showed that As(III) was more detrimental to plant growth than As(V) in terms of plant growth, biomass, and lipid peroxidation in both chronic and acute exposure. Furthermore, overexpression of *OsGSTU40* led to better plant growth even though uptake of As(V), but not As(III), into shoots was enhanced in transgenic plants. In acute As(III) stress, transgenic plants exhibited a lower level of lipid peroxidation than wild-type plants. The element composition of plants was dominated by the different As stress treatments rather than by the genotype, while the As concentration was negatively correlated with phosphorus and silicon. Overall, our findings suggest that As(III) is more toxic to plants than As(V) and that glutathione S-transferase *OsGSTU40* differentially affects plant reactions and tolerance to different species of arsenic.

**Supplementary Information:**

The online version contains supplementary material available at 10.1007/s11356-023-28833-x.

## Introduction

Arsenic (As) is a toxic metalloid widely found in nature and categorized as a class-1 human carcinogen (Cancer 2012), posing a potential health risk to humans and animals through dietary consumption (Meharg et al. 2009; Zhao et al. 2010). Rice, which is a staple crop feeding more than half of the world’s population (IRRI 2016), often takes up and accumulates large amounts of As from soils (Ma et al. 2008; Sohn 2014; Williams et al. 2007; Zhao et al. 2010). Many paddy soils in Asian countries such as Bangladesh, China including Taiwan, India, Japan, Korea, Laos, Myanmar, Nepal, Pakistan, the Philippines, and Vietnam, i.e., major rice-growing areas in the world contain high amounts of As in soil and groundwater (Shaji et al. 2021; Williams et al. 2005). Globally, an estimated 220 million people are potentially exposed to high concentrations of arsenic (Podgorski and Berg 2020). In the Ganges–Brahmaputra deltas of India and Bangladesh, specifically, approximately 40 million individuals are directly exposed to this hazardous class I carcinogen (Rahaman et al. 2013). In this region, the average concentration of arsenic in agricultural topsoil has been reported to range from 1.5 to 19 mg kg^−1^, with levels reaching as high as 83 mg kg^−1^ in heavily contaminated areas (Saha and Rahman 2020). Arsenic is carried by rivers from Himalayan rock sediments to rice-producing regions of South and Southeast Asia (Benner and Fendorf 2010). Irrigation of fields with As-contaminated groundwater decreases crop yields and poses risks to human health (Brammer 2009; Williams et al. 2006; Zhao et al. 2015). Rice plants cultivated in such environments inevitably take up elevated amounts of As through roots and further transfer it to shoots (Zhao and Wang 2020). Consumption of such products increases the risk of As toxicity in humans (Li et al. 2011; Meharg et al. 2009; Stone 2008; Zhao et al. 2010).

Recent increases in CO_2_ levels are driving temperature changes and affecting soil biogeochemical processes by altering microbial community dynamics and geochemical reactions that include contaminant absorption and desorption (Castro et al. 2010; Compant et al. 2010; Frey et al. 2008). These biogeochemical soil cycles are likely to exacerbate the problems of As accumulation in rice grains (Muehe et al. 2019). Furthermore, high As uptake is detrimental to plant growth, development, and reproduction, leading to substantial yield losses (Huhmann et al. 2017; Mishra et al. 2021; Panaullah et al. 2009; Wang et al. 2020). Therefore, it is essential to develop resistant cultivars that accumulate less As in the grains. To prevent the accumulation of As in rice shoots and grains posing potential health risks to humans from rice consumption, it is essential to understand the mechanisms of As uptake, translocation, and accumulation in rice. This requires a focus on arsenic speciation and availability.

Arsenic occurs in two major chemical groups, organic and inorganic. Organic forms of As, e.g., methylated As present in plant and animal cells, are less toxic, whereas inorganic forms of As are highly toxic and cause significant health hazards. The prevalent forms of inorganic arsenic species are arsenite (As(III)) and arsenate (As(V)), which differ in their mobility, bioavailability and toxicity. The speciation of arsenic is governed by the physicochemical conditions of the environment, especially soil pH and redox potential (Wang et al. 2020). Under aerobic conditions, As(V) is dominant (Ding et al. 2017; Huang et al. 2011; Jia et al. 2014), while the proportion of As(III) increases in anaerobic environments (Ma et al. 2008; Zhao et al. 2013). Since soil conditions in rice fields switch between dry and wet, both forms of arsenic are present in field conditions depending on the soil redox potential.

Plant roots take up As(V) from soils mainly by phosphate transporters (Cao et al. 2017; Wang et al. 2016), whereas silicon transporters mediate As(III) uptake (Chen et al. 2017; Xu et al. 2015). Although the modes of toxicity are different for different species, both As(V) and As(III) are toxic to plant cells. Arsenic alters various metabolic pathways in plant cells. As(V) replaces phosphate in several reactions, hampering oxidative phosphorylation and interrupting glucose and energy metabolism (Finnegan and Chen 2012). In contrast, As(III) reacts with the thiol group of cysteines and thereby altering the activity of respective proteins (Shen et al. 2013). Exposure of plants to As reduces the photosynthesis rate, alters carbohydrate metabolism, induces oxidative stress through reactive oxygen species (ROS) generation, and causes lipid peroxidation (Gupta et al. 2013). Plants possess cellular mechanisms to overcome such stress and detoxify the effect of As. One such mechanism for As detoxification in plant cells at the biochemical level is chelation with thiol compounds such as glutathione (GSH) or phytochelatin (PC), subsequently reducing As mobility and sequestering it into the vacuole (Raab et al. 2005; Song et al. 2010). In addition, antioxidant enzymes such as superoxide dismutase, catalase, and glutathione S-transferase (GST), as well as ascorbate, are involved in ROS detoxification (Hartley-Whitaker et al. 2001, Mylona et al. 1998). In addition, GST enzymes catalyze the formation of As(III)-glutathione conjugates and transport the conjugates into the vacuole (Kumar and Trivedi 2018). Although As stress in rice plants has been studied extensively, the differential effects of different As species on plant performance and stress responses remain poorly understood.

Glutathione S-transferases (GSTs, EC 2.5.1.18) are a group of multifunctional enzymes that provide tolerance against various abiotic (Kumar et al. 2013) and biotic stresses (Ding et al. 2017), such as heavy metals (Tiwari et al. 2022; Tripathi et al. 2014), pesticides (Aioub et al. 2021), ultraviolet radiation (Liu and Li 2002), and pathogen attacks (Tiwari et al. 2020). GSTs are associated with plant developmental processes and responses to many stressors by quenching reactive molecules with the addition of glutathione and protecting cells from oxidative damage (Kumar and Trivedi 2018). These enzymes catalyze the conjugation of the reduced form of GSH to a mixture of electrophilic and hydrophobic substrates, forming GSH complexes for detoxification (Aioub et al. 2023). The GSH-conjugated compounds become more water-soluble and are further sequestered into the vacuole, thereby reducing the translocation to other parts of plant (Labrou et al. 2015). If sequestration and accumulation of As occur in the vacuole of roots, translocation of toxic compounds to the shoot is prevented. Based on phylogeny, plant GSTs are classified into seven classes, among which members of the phi- and tau-classes are reported to be involved in detoxifying xenobiotic compounds (Soranzo et al. 2004; Tiwari et al. 2022). Several tau-class GSTs from rice were reported to provide tolerance against various heavy metals (Tiwari et al. 2022).

Microarray analysis of *japonica* and *indica* subspecies of rice by Norton et al. (2008) found that the transcripts of *LOC_Os01g49710* encoding *OsGSTU40* are upregulated with As treatment. Similar results were also reported in previous transcriptome analyses of the major rice variety IR64 under As(III) and As(V) stress, which showed higher expression of *OsGSTU40* under As(V) toxicity (Chakrabarty et al. 2009). A similar transcriptional regulation of *OsGSTU40* was reported with other metal toxicities (Matthus et al. 2015). In a previous genome-wide association study (GWAS) on the tolerance mechanisms to iron (Fe) toxicity in rice, *OsGSTU40* was identified within a tolerant locus on chromosome 1 associated with foliar symptoms (Matthus et al. 2015) and proposed as a candidate gene for Fe toxicity tolerance. However, the role of the gene in tolerance to any element toxicity has not been confirmed by a reverse genetic approach. Thus, evidence for its involvement in metal tolerance remains circumstantial.

In this study, we evaluated the effects of different concentrations of As(III) and As(V) representing acute or chronic arsenic stress on wild-type and *OsGSTU40* overexpression plants. We address two research objectives: (1) to differentiate rice plant responses to As(III) and As(V) at different concentrations based on physiological and biochemical analyses and (2) to evaluate the roles of *OsGSTU40* in As stress tolerance. These efforts are expected to enhance our mechanistic understanding of the As stress response and tolerance and aid in devising specific adaptation strategies for different environmental conditions.

## Materials and methods

### Overexpression vector construction and rice transformation

To generate *OsGSTU40*-overexpressing lines, the coding sequence (CDS, 738 bp) of *OsGSTU40 (LOC_Os01g49710)* was first synthesized by GeneScript (GenScript Biotech Corporation, Rijswijk, Netherlands) and cloned into a Gateway™ donor vector pDONR201, resulting in an entry vector. The *OsGSTU40* CDS was further introduced into the destination vector pMBb7Fm21GW-UBIL through an LR reaction using LR Clonase™ II (Thermo Fisher Scientific, Schwerte, Germany). The final expression vector pZmUBIL::*OsGSTU40* (Supplementary Fig. S1) was introduced into *Agrobacterium tumefaciens* EHA105 cells through electroporation.

Rice transformation was performed according to Ji et al. (2015). In brief, healthy seeds of Nipponbare (*Oryza sativa* L., ssp. *japonica*) were dehusked and disinfected using 70% ethanol (5 min) and NaClO solution (with 14% active chlorine; 30 min). After thoroughly rinsing with autoclaved ddH_2_O, seeds were placed on N6 callus induction medium containing 2 mg/L 2,4-dichlorophenoxyacetic acid (2,4-D) at 28 °C in the dark. The generated embryogenic calli were inoculated with Agrobacterium culture harboring the overexpression vector (OD600 = 0.3–0.5). Inoculated calli were selected with 50 mg/L glufosinate (Sigma‒Aldrich, Taufkirchen, Germany). Newly generated healthy calli were subsequently transferred to shoot and root regeneration medium to obtain functional plantlets. The regenerated T_0_ plants were grown in a glasshouse under the following conditions: light/dark period, 14/10 h; photosynthetic photon flux density (PPFD), 400–700 µmol m^−2^ s^−1^; day/night temperature, 28 °C/22 °C, relative humidity, 60–70%.

To obtain transformed plants with single-copy insertion, genomic DNA from 16 T_1_ seedlings were isolated using Plant DNA mini kit (peqGOLD, VWR International, Radnor, USA). Genotyping PCR were conducted with a pair of primers targeting the Bialaphos resistance gene, *bar* (primer sequences listed in Supplementary Table S3). The genotyping results were subjected to a *χ*^2^ test (*p* = 0.05). Two overexpression (OE) lines showing a ratio of 3 (positive): 1 (negative) were selected for seed propagation (Ali et al. 2019). Seeds from homozygous T_3_ plants OE #2 and OE #8 carrying single-copy insertion were used in the screening experiment.

### Phylogeny analysis

Protein sequences of 82 rice *GST* genes were obtained from the Rice Genome Annotation Project (RGAP 7, http://rice.uga.edu/index.shtml) and Uniprot (www.uniprot.org/). Multi-alignment of the protein sequences was performed using the Clustal Omega program (https://www.ebi.ac.uk/Tools/msa/clustalo/). To remove the poorly aligned regions, aligned sequences were trimmed with trimAI (Capella-Gutiérrez et al. 2009). Phylogenetic tree was generated with FASTME 2.0 using a distance-based approach (FastME distance matrix). Clade-supporting scores were calculated by 500 bootstrapping replicates, and the output phylogenetic tree was visualized using iTol (https://itol.embl.de/).

### Plant materials and growth conditions

Screening experiments were performed using wild-type (WT) Nipponbare and two *OsGSTU40* OE lines (OE #2, OE #8). Dehusked seeds were surface sterilized with 70% ethanol and 10% NaClO solution, followed by thorough rinses with autoclaved ddH_2_O. Disinfected seeds were placed on half-strength Murashige and Skoog (½MS) medium (pH 5.8) in darkness at 30 °C for five days and transported to light conditions for two days. All emerged seedlings of OE lines and 10 WT (as negative control) seedlings were transferred to medium containing 50 mg/L antibiotic glufosinate. OE seedlings showed a 100% survival rate, and all WT seedlings died after one week of selection. Two-week-old seedlings were transplanted into containers (20 L) filled with quarter-strength Yoshida nutrient solution (pH 5.5). The nutrient solution was changed to half-strength five days after transplanting. After growing the seedlings in the half-strength nutrient solution for one week, full strength was used to acclimatize the young plants. The full composition of the full-strength nutrient solution is shown in Supplementary Table S1. The experiment was performed in a fully climate-controlled growth chamber at the University of Giessen (Germany) with the following settings: day/night length, 8/16 h; PPFD, 600–800 µmol m^−2^ s^−1^; day/night temperature, 28 °C/22 °C; relative humidity, 60–70%. The plants were grown until 26 days old before the As treatment. All experiments were conducted with four replicates, and the pH of the nutrient solutions was adjusted to 5.5 daily.

### Hydroponic experiments and arsenic treatment

Plants were subjected to two types of stress: acute stress, where they were exposed to 10 mg L^−1^ arsenic for ten days, and chronic stress, where they were exposed to 2 mg L^−1^ arsenic for 20 days. In each stress condition, two different inorganic arsenic species were separately used to induce the stress. For As(III), sodium arsenite (NaAsO_2_) was employed, while for As(V), sodium arsenate dibasic heptahydrate (Na_2_HAsO_4_.7H_2_O) was used. A total of 12 containers were set up for each stress condition, with three treatments (control, As(III), and As(V)) and four replicates. Three individual plants of each genotype were grown in one container. After As application, the solution in chronic stress was renewed once in 10 days. The nutrient solution was not aerated during the experimental period to mimic anaerobic conditions, during which period arsenic species were stable in the nutrient solution. At the end of the experiment, shoot and root tissues were separately collected for further analyses.

### Phenotypic analysis

Shoot and root lengths were measured on the day of harvest. Shoot and root dry weights were determined after drying samples for at least three days at 60 °C. Genotypic responses to stress conditions for the treatment and individual genotypes were photographed using a digital camera (Sony, ILCE-7M3). Growth parameters were compared between WT and two independent OE lines in different treatments.

### Biochemical assays

Twenty-six-day-old plants were subjected to acute and chronic As(III) or As(V) stress. After the treatment, shoot and root tissues were harvested separately from each genotype into liquid nitrogen and stored at − 80 °C for further analyses.

MDA (malondialdehyde) was measured according to Höller et al. (2015). One hundred milligrams of samples were extracted with 1.5 ml of 0.1% (w/v) trichloroacetic acid (TCA). After centrifugation at 14,000 × g for 15 min at 4 °C, clear supernatants were collected to prepare two aliquots. Aliquots were separately mixed with two reaction solutions containing 20% (w/v) TCA and 0.01% (w/v) 2,6-di-tert-butyl-4-methylphenol with and without an additional 0.65% (w/v) thiobarbituric acid (TBA). The mixture was heated at 95 °C for 30 min and then transferred to ice for 5 min to stop the reactions. The absorbance at 440, 532, and 600 nm were measured. Blank samples were prepared with 0.1% (w/v) TCA solution instead of the sample, and the absorbance was subtracted from each sample value.

GST activity was determined by measuring the rate of conjugation of GSH to 1-chloro-2,4-dinitrobenzene (CDNB) to form the GSH-CDNB complex in the presence of enzyme extract (Habig et al. 1974). Enzyme extracts were obtained from 50 mg of frozen shoot samples mixed with 1 ml of extraction buffer containing 100 mM Tris–HCl, pH 7.5, 2 mM EDTA, and 1 mM dithiothreitol (DTT). The homogenate was vortexed vigorously for 30 s and centrifuged at 15,000 × g for 15 min at 4° C. A reaction mixture containing 70 µL of assay buffer (100 mM potassium phosphate buffer with 1 mM EDTA, pH 6.5), 10 µL of enzyme extract, 10 µL of 10 mM GSH, and 10 µL of 10 mM CDNB was prepared, and the absorbance of the conjugated product was measured at 340 nm for 10 min (*ε* = 9.6 mM^−1^ cm^−1^) using a microplate reader (Infinite 200 pro, Tecan, Groedig, Austria). The total protein concentration was measured using Bradford reagent (Sigma) (Bradford 1976).

### Element analysis

Two plants from each genotype in each treatment were separated into roots and shoots, carefully washed with ddH_2_O, and dried at 65 °C for three days. Dried shoot and root tissues were ground to fine powders, and approximately 300 mg of shoot samples and 200 mg of root samples were digested with HNO_3_ (65%) using a microwave oven (Anton Paar, Microwave reaction system, Multiwave 5000, Austria). Reagent blanks and certified reference materials (Green Tea NCS ZC73036a from NCS Testing Technologies Co., Ltd., Beijing, China) were included for quality control in the analysis. After digestion, the solutions were adjusted to 25 mL with Milli-Q water and filtered. The concentrations of arsenic, phosphorus, silicon, and other mineral elements in the shoots and roots of the acid digests were determined using inductively coupled plasma‒mass spectrometry (iCAP PRO ICP OES Duo, Thermo Fisher Scientific, USA).

### RNA extraction and quantitative real-time PCR

Total RNA was extracted from shoot tissue using the RNeasy Plant Mini Kit (Qiagen) following the manufacturer’s protocol. On-column DNase treatment was performed with an RNase-Free DNase Set (Qiagen) to remove genomic DNA contamination. One and a half micrograms of total RNA was used for first-strand cDNA synthesis (iScriptTM gDNA Clear cDNA Synthesis Kit, Bio-Rad) following the manufacturer’s instructions. Control reactions were performed without the reverse transcriptase enzyme to verify that no DNA contamination was present in the RNA samples.

Quantitative real-time PCR was performed on a StepOnePlus qPCR machine (Applied Bioscience, USA) using a SYBR Green Master Mix kit (Promega) according to the manufacturer’s instructions. The rice *ubiquitin 5* (*UBQ5*) gene was used as the internal reference. The expression level of each gene was calculated as 2^−ΔΔCt^ relative to the internal reference. The primers used are listed in Supplementary Table S2.

### Statistical analysis

For statistical analysis, data were checked for normality, and data below and above 1.5 times the interquartile range were removed for further analysis. One-/two-way ANOVA and Dunnett’s post hoc test were used to determine statistical significance (*P* < 0.05) between the control and the overexpressed plants for all the parameters. ANOVA was also used to test the significant difference between different treatments. R program version 4.1.1 and the packages dplyr, nmle, emmeans, multcomp, and ggplot2 were used for analysis and generating graphs (R core Team 2021). Principal component analysis (PCA) was performed to observe the pattern of variation in element uptake using JMP® software.

## Results

### *LOC_Os01g49710*, a member of tau-class glutathione S-transferases

**Fig. 1:**
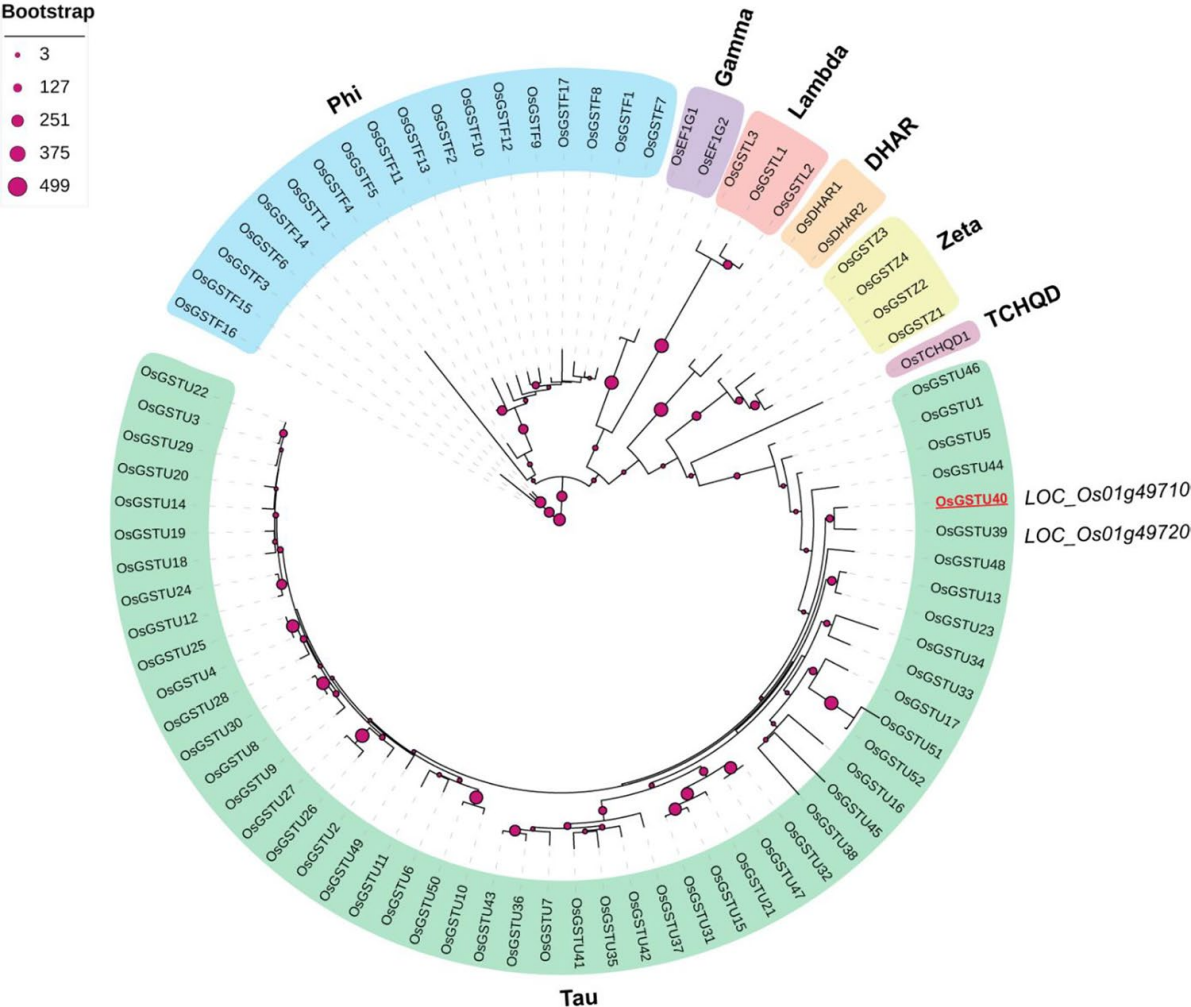
Phylogeny of glutathione S-transferase genes. Phylogenetic trees of rice glutathione S-transferase genes were generated using protein sequences with the help of NGPhylogeny (https://ngphylogeny.fr/) and visualized with the iTOL program (https://itol.embl.de/). In total, seven clusters were identified among 82 rice genes: tau, phi, gamma, lambda, DHAR, zeta, and TCHQD. *LOC_Os01g49710* was identified as *OsGSTU40* in the tau-class, which is marked in red and underlined. Clade clustering scores shown as the bootstrap replicates were calculated based on bootstrapping (*n* = 500). The number of bootstrap replicates is represented by different sizes of blue cycles

Phylogenetic analysis indicated that *LOC_Os01g49710* belongs to the largest tau-class of the rice glutathione S-transferase gene family (Fig. 1). Hereafter, we designated *LOC_Os01g49710* as *OsGSTU40.* The closest paralog of this gene is *LOC_Os01g49720* (*OsGSTU39*). Both genes show a conserved exon–intron structure (Supplementary Fig. S2A), indicating that these two genes might have arisen from recent tandem duplication. The transcript level was most abundant in the panicles at the reproductive stage, especially in the inflorescence at the P6 stage and young seed at the S1 stage (Supplementary Fig. S2B) (Waese et al. 2017). Abiotic stresses such as cadmium, dehydration, cold, and osmotic and abscisic acid (ABA) highly induced its mRNA level in both root and shoot tissues (Supplementary Fig. S3). Under As(III) stress, *OsGSTU40* transcript levels were suppressed in shoots but largely induced in roots (Yu et al. 2012). Similar results were reported by Norton et al. (2008), who showed that *OsGSTU40* transcription in rice roots was induced by As(V) treatment.

### *OsGSTU40* OE lines are more tolerant to high concentrations of As

**Fig. 2:**
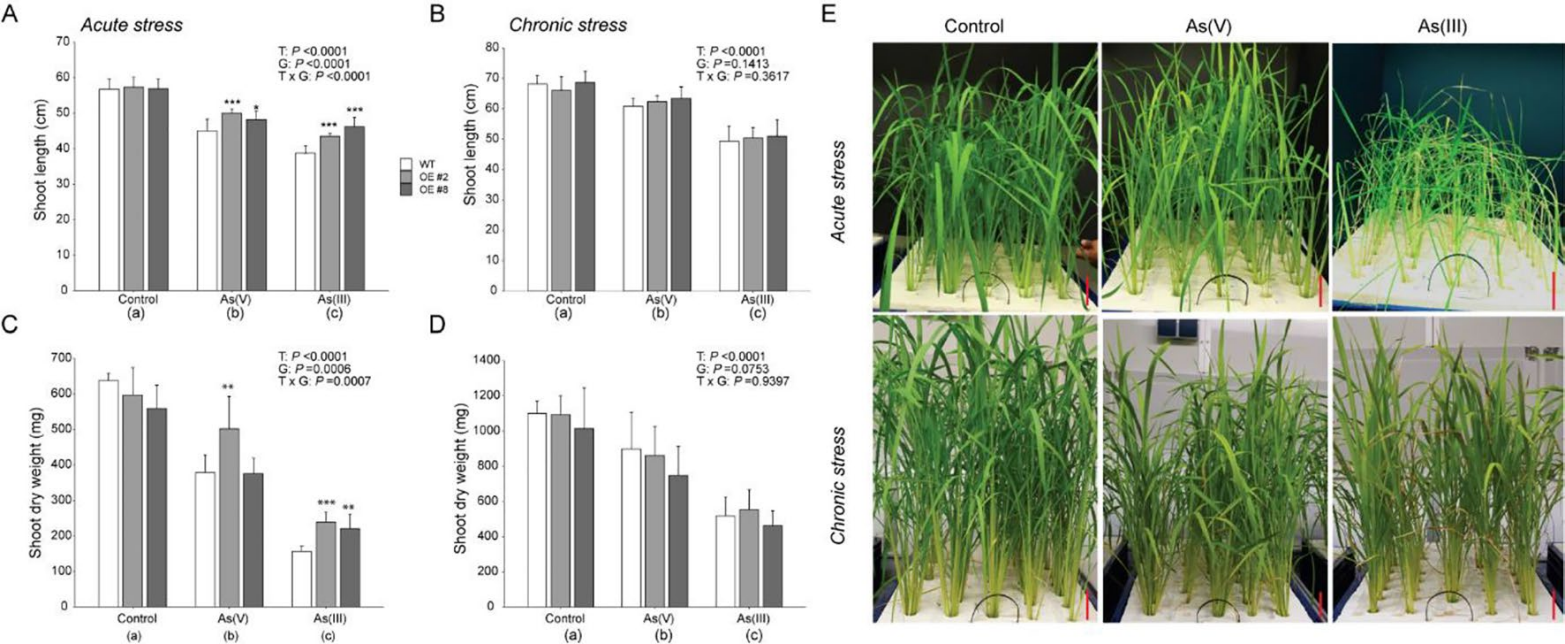
Growth phenotype of Nipponbare (WT) and *OsGSTU40* OE lines. **A** Shoot length under acute stress. **B** Shoot length under chronic stress. **C** Shoot biomass under acute stress. **D** Shoot biomass under chronic stress of WT and OE rice plants grown hydroponically under different species and concentrations of arsenic. **E** Effect of different arsenic treatments on plant growth. Acute stress: 10 mg L^−1^ arsenic for 10 days; chronic stress: 2 mg L^−1^ arsenic for 20 days. Data are from 4 biological replicates, and the bar represents the mean ± SD (*n* = 12). Significant differences between WT and OE lines were calculated using Dunnett’s test indicated by *P < 0.05, **P < 0.01, or ***P < 0.001. The letters below the treatments in parentheses indicate significant differences between different treatment conditions. As(V): sodium arsenate dibasic heptahydrate; As(III): sodium arsenite; T: treatment; G: genotype; T × G: treatment by genotype interaction. Scale bars in **E** represent 5 cm

Twenty-six-day-old WT, OE #2, and OE #8 plants were treated with different concentrations of As(III) and As(V), and the roles of *OsGSTU40* in combating As stress were analyzed. Under control conditions (without As), the two OE lines showed similar growth compared to the WT. Under As(III) and As(V) acute stress treatment, both OE lines showed higher levels of shoot length than WT. WT showed 32% and 21% reductions in shoot length, whereas OE lines only showed 20% and 16% reductions caused by As(III) and As(V) acute stress, respectively (Fig. 2 A). However, under chronic stress (2 mg L^−1^ As for 20 days) conditions, no genotypic differences were observed for the shoot length between WT and OE lines regardless of As species. Strikingly, the shoot length of all genotypes was decreased more significantly by As(III) than by As(V), indicating that As(III) is more detrimental to rice plants. Across all genotypes, shoot length reduction was 25% in the As(III) treatment and ~ 10% in the As(V) treatment compared to the control conditions (Fig. 2 B).The root length decreased in response to As treatment under both acute and chronic stress (Table 1, Supplementary Fig. S4). Overall, a 30% reduction in root length was observed in the acute stress treatment, whereas in the chronic stress treatment, As(III) and As(V) led to 20% and 7% reductions in root length, respectively. Maximum tolerance in terms of root length was observed in OE lines in As(III) treatment under both acute and chronic stress (Table 1).

**Table 1:** Morphological traits of Nipponbare (WT) and *OsGSTU40* OE rice plants. The root traits were measured in the vegetative growth phase of plants after application of As for 10 and 20 days for acute and chronic stress, respectively

Treatment/genotype	Root length (cm)		Root biomass (mg)	
	10 mg L^−1^	2 mg L^−1^	10 mg L^−1^	2 mg L^−1^
**Control**	**a**	**a**	**a**	**a**
WT	18.3 ± 1.1	22 ± 2.3	181 ± 22	230 ± 18
OE #2	18.5 ± 1.6	23 ± 1.9	163 ± 16	252 ± 48
OE # 8	19.1 ± 1.4	21.6 ± 0.7	154 ± 41	215 ± 46
**As(V)**	**b**	**b**	**b**	**a**
WT	12.5 ± 1.4	20.3 ± 2.2	116 ± 35	245 ± 63
OE #2	13.9 ± 0.9	21.1 ± 2.5	133 ± 11	192 ± 34
OE #8	12.8 ± 1.7	20.6 ± 1.8	119 ± 25	195 ± 32
**As(III)**	**b**	**c**	**c**	**b**
WT	12.3 ± 0.8	17.1 ± 1.6	41 ± 6	187 ± 37
OE #2	14.5 ± 1.3***	18.7 ± 0.8**	48 ± 5	182 ± 18
OE #8	13.8 ± 1.2**	18.6 ± 0.5*	44 ± 7	152 ± 25

*As(III)* sodium arsenite, *As(V)* sodium arsenate dibasic heptahydrate

Acute stress: 10 mg L^−1^ ppm for 10 days; chronic stress: 2 mg L^−1^ for 20 days

Data are from 4 biological replicates, values representing means ± SDs (*n* = 12 for all traits and *n* = 8 for root biomass)

Significant differences between WT and OE lines were calculated using Dunnett’s test indicated by *P < 0.05, **P < 0.01, or ***P < 0.001

Alphabets in treatment rows are the indication of significant differences between different treatment conditions

As stress significantly reduced the shoot biomass of all genotypes, but the reduction was less pronounced for OE lines than for WT in acute As treatment. For WT, shoot biomass was reduced 75% and 40% by acute stress of As(III) and As(V), respectively, whereas only 40% and 25% reduction were observed in OE lines compared to the control treatment (Fig. 2 C). Overall, shoot biomass was reduced by As chronic stress treatment, but significant differences between the OE lines and WT were not observed (Fig. 2 D).

Since roots are the entry site for As transport into the plant, root biomass is an important parameter characterizing the toxic effect of As. In the acute As treatment, root biomass was significantly reduced for all genotypes. Chronic As(V) treatment did not reduce root biomass significantly, in contrast to the As(III) treatment, which largely reduced root biomass in all genotypes (Table 1). In summary, overexpression of *OsGSTU40* helped rice plants maintain shoot and root development under acute As stress.

### Transcript abundance of *OsGSTU40* varied with As concentration and species

**Fig. 3:**
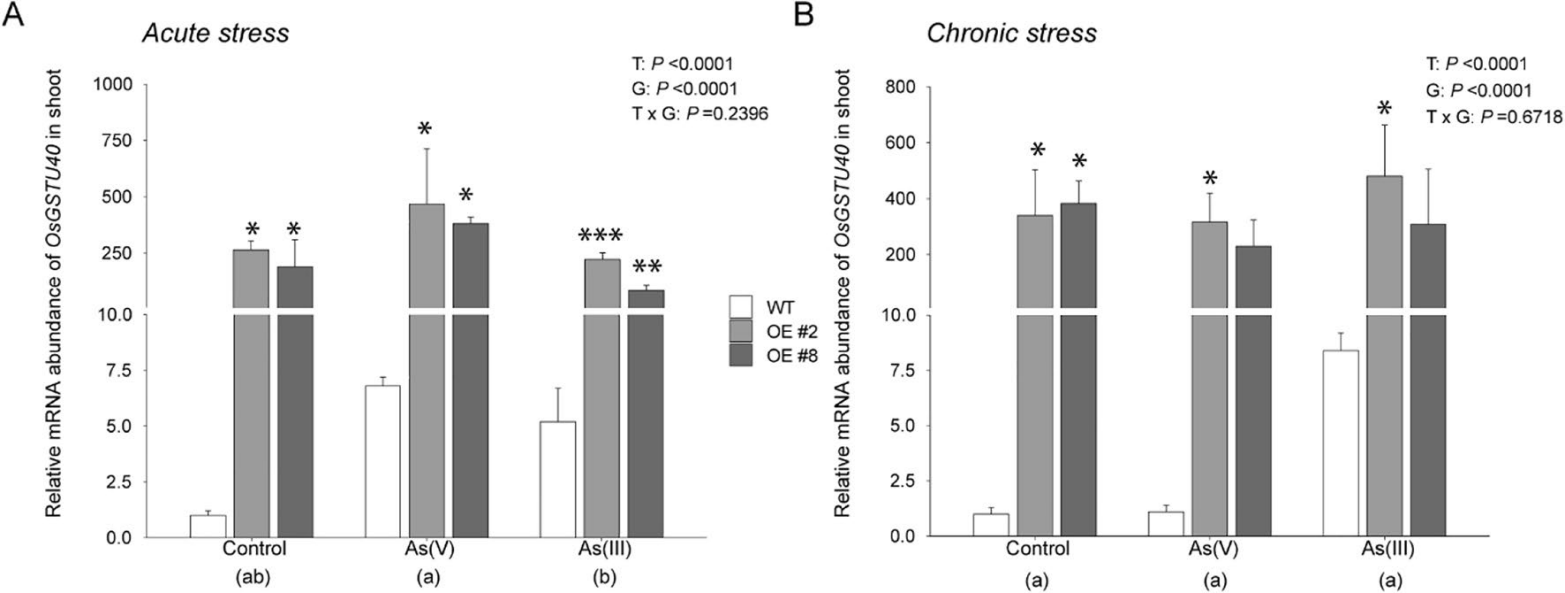
Expression analysis of the *OsGSTU40* gene in Nipponbare (WT) and *OsGSTU40* OE lines. Real-time PCR analysis of WT and OE rice plants differing in the *OsGSTU40* gene grown hydroponically under different species and concentrations of arsenic: **A** acute stress and **B** chronic stress. Acute stress: 10 mg L^−1^ arsenic for 10 days; chronic stress: 2 mg L^−1^ arsenic for 20 days. The rice *ubiquitin 5* (*UBQ5*) gene was used as a control to estimate relative expression. The mRNA levels for different rice lines were calculated relative to their expression in the control plant Nipponbare. Data are from 3 biological replicates, and the bar represents the mean ± SD (*n* = 3). Significant differences between WT and OE lines were calculated using Dunnett’s test indicated by *P < 0.05, **P < 0.01, or ***P < 0.001. The letters below the treatments in parentheses indicate significant differences between different treatment conditions. As(V): sodium arsenate dibasic heptahydrate; As(III): sodium arsenite; T: treatment; G: genotype; T × G: treatment by genotype interaction

The expression pattern of *OsGSTU40* was investigated in rice lines treated with different As species under two types of stresses, acute and chronic. As predicted, OE lines showed significantly higher (200-fold) transcript levels of *OsGSTU40* than WT under control conditions (Fig. 3 A and B).

Furthermore, we investigated the effect of As treatment on *OsGSTU40* transcript regulation. Under acute stress of As(III) and As(V), the expression of *OsGSTU40* in WT was increased by fourfold compared to the control treatment, whereas expression increased in OE lines by As(V) but decreased by As(III) when compared to the OE lines in the control treatment. In both treatments, the transcript level was significantly higher in OE lines than in WT lines in all treatments (Fig. 3 A). Under chronic stress, *OsGSTU40* transcription in WT was strongly induced only by As(III), with an eightfold increase (Fig. 3 B). In contrast to the acute stress treatment, *OsGSTU40* transcription in the OE line increased in the As(III) treatment and decreased in the As(V) treatment compared to the control conditions (Fig. 3 B).

### *OsGSTU40* overexpression increases GST activity during As(III) treatment

**Fig. 4:**
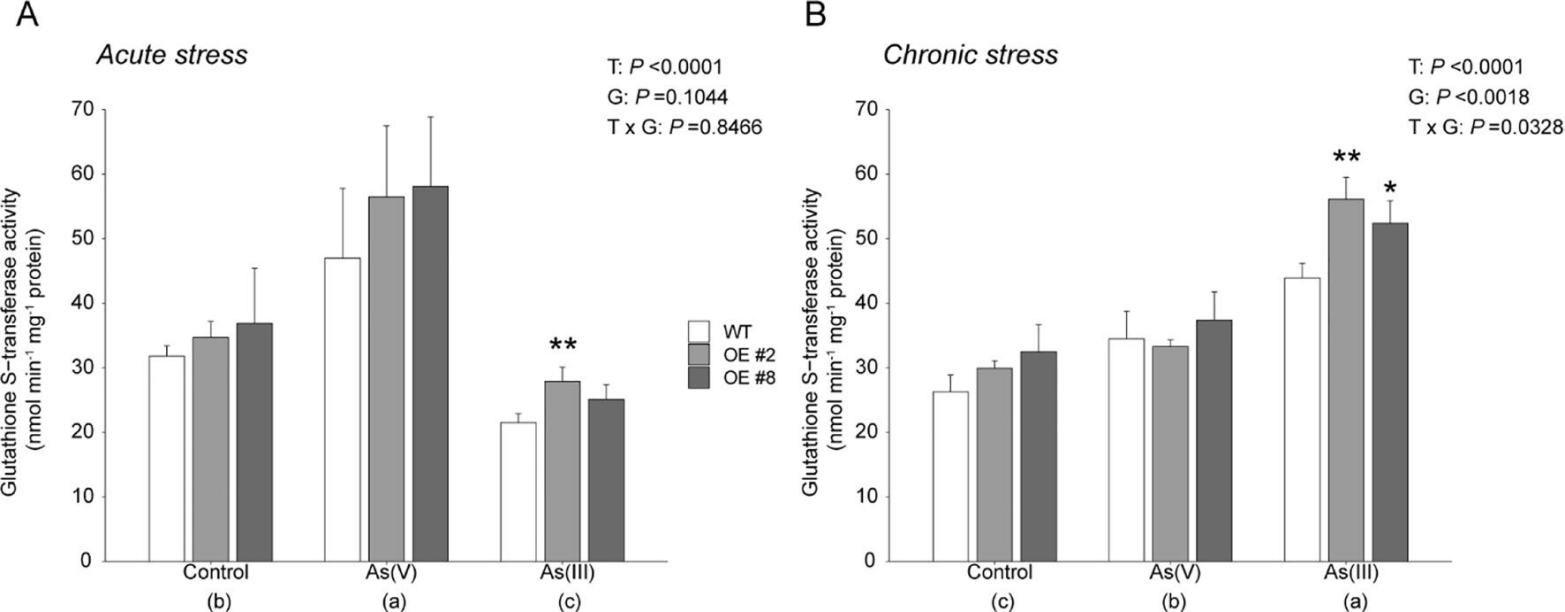
Analysis of GST activity in Nipponbare (WT) and *OsGSTU40* OE lines. GST activity of WT and OE rice plants differing in the *OsGSTU40* gene grown hydroponically under different species and concentrations of arsenic: **A** acute stress and **B** chronic stress. Acute stress: 10 mg L^−1^ arsenic for 10 days; chronic stress: 2 mg L^−1^ arsenic for 20 days. Protein concentration measured based on bovine serum albumin (BSA) activity. Data are from 4 biological replicates, and the bar represents the mean ± SD (*n* = 4). Significant differences between WT and OE lines were calculated using Dunnett’s test indicated by *P < 0.05, **P < 0.01, or ***P < 0.001. The letters below the treatments in parentheses indicate significant differences between different treatment conditions. As(V): sodium arsenate dibasic heptahydrate; As(III): sodium arsenite; T: treatment; G: genotype; T × G: treatment by genotype interaction

Both WT and OE lines were analyzed to estimate the contribution of *OsGSTU40* transcription to overall GST activity. The OE lines showed 10–30% more GST enzyme activity than the WT under all treatment conditions (Fig. 4 A and B). However, GST activity in all genotypes was increased by acute stress of As(V) and decreased by acute As(III) treatment (Fig. 4 A). In contrast, under chronic stress, higher GST activity was found in plants treated with As(III) (Fig. 4 B). GST activity was significantly higher in OE lines when treated with As(III) under both acute and chronic stress conditions. Altogether, these data revealed that the increased GST activity in OE lines correlated with the overexpression of *OsGSTU40*.

### *OsGSTU40* overexpression reduced lipid peroxidation in As(III) treatment

**Fig. 5:**
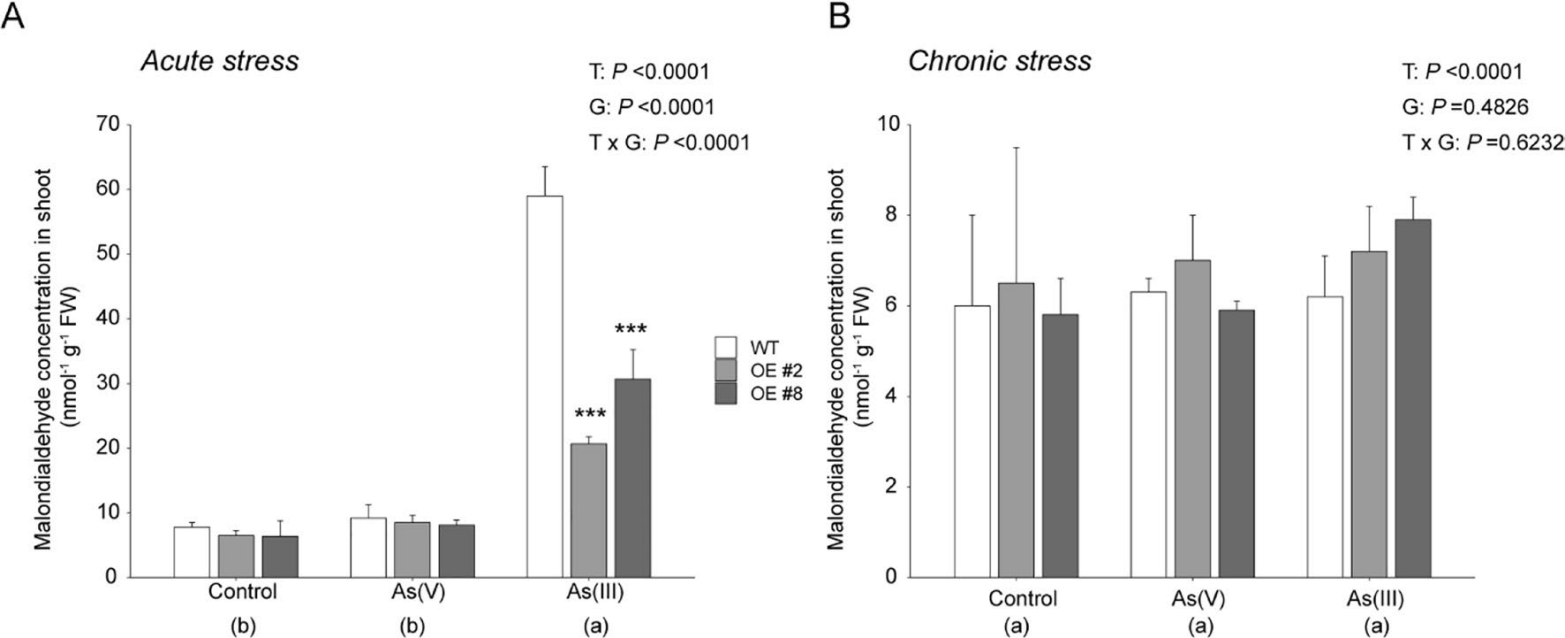
Lipid peroxidation status in WT (Nipponbare) and *OsGSTU40* OE lines. Malondialdehyde content of WT and OE rice plants differing in the *OsGSTU40* gene grown hydroponically under different species and concentrations of arsenic: **A** acute stress and **B** chronic stress. Acute stress: 10 mg L^−1^ arsenic for 10 days; chronic stress: 2 mg L^−1^ arsenic for 20 days. Data are from 4 biological replicates, and the bar represents the mean ± SD (*n* = 4). Significant differences between WT and OE lines were calculated using Dunnett’s test indicated by *P < 0.05, **P < 0.01, or ***P < 0.001. The letters below the treatments in parentheses indicate significant differences between different treatment conditions. As(V): sodium arsenate dibasic heptahydrate; As(III): sodium arsenite; T: treatment; G: genotype; T × G: treatment by genotype interaction

The MDA concentration in shoots was analyzed to determine the oxidative stress caused by As(III) and As(V) treatment in rice plants. In acute As treatments, only As(III) significantly induced shoot MDA concentration, while no treatment effect was observed in the As(V) treatment. The OE lines showed approximately 50% lower MDA concentrations than the WT (Fig. 5 A). Under chronic stress conditions, no significant increases in shoot MDA were observed in all lines (Fig. 5 B). Higher transcript levels of *OsGSTU4*0 in OE lines correlated with lower lipid peroxidation under acute As(III) stress conditions. The chronic stress of both As(III) and As(V) did not change the lipid peroxidation status of plant tissues (Fig. 5 A and B).

### *OsGSTU40* overexpression affects As accumulation

**Fig. 6:**
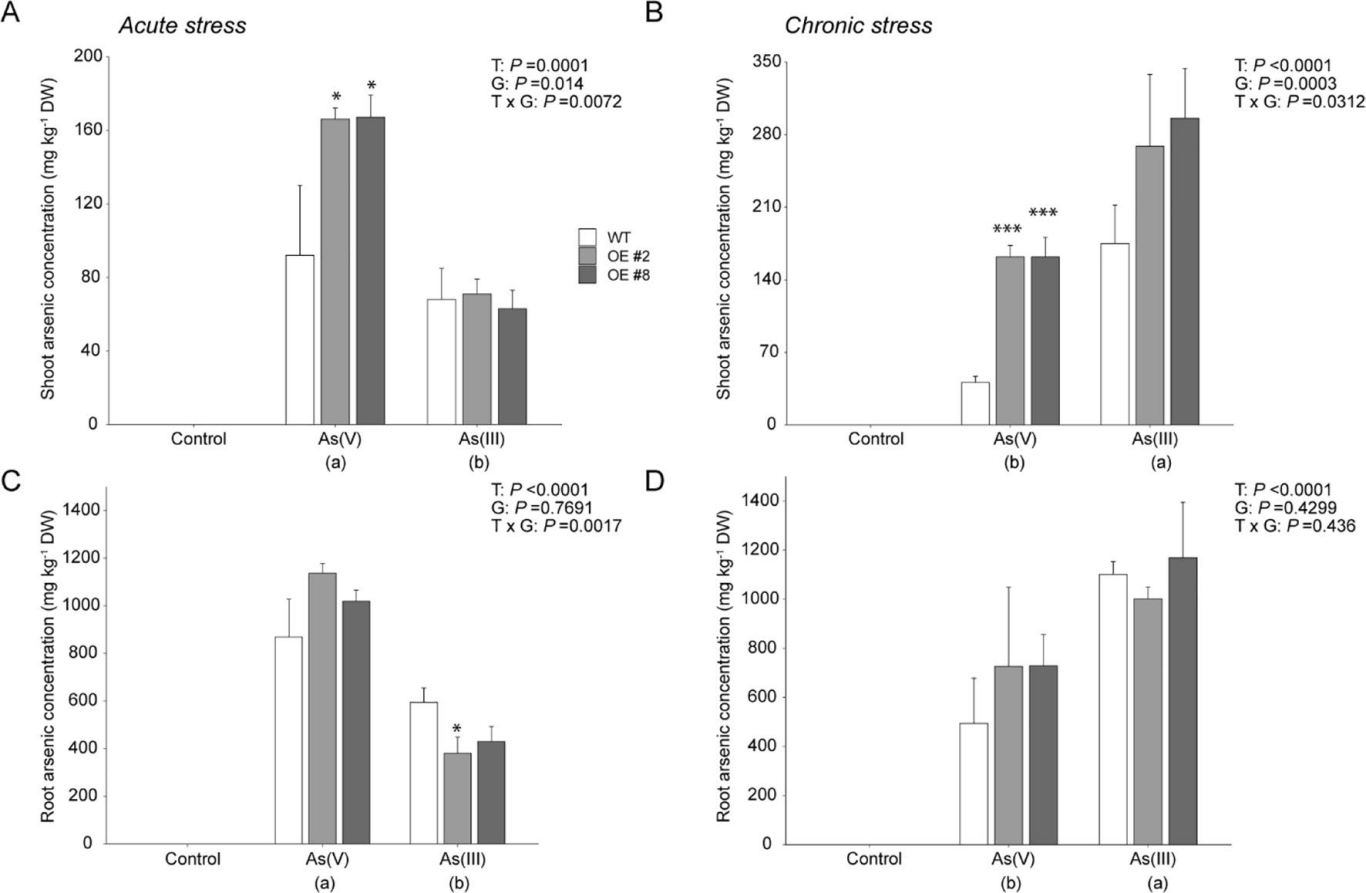
Arsenic accumulation in shoots and roots. Arsenic concentration in WT and OE rice plants differing in the *OsGSTU40* gene and grown hydroponically under different species and concentrations of arsenic. **A** Shoot arsenic content under acute stress. **B** Shoot arsenic content under chronic stress. **C** Root arsenic content under acute stress. **D** Root arsenic content under chronic stress. Acute stress: 10 mg L^−1^ arsenic for 10 days; chronic stress: 2 mg L^−1^ arsenic for 20 days. Data are from 3 biological replicates, and the bar represents the mean ± SD (*n* = 6). Significant differences between WT and OE lines were calculated using Dunnett’s test indicated by *P < 0.05, **P < 0.01, or ***P < 0.001. The letters below the treatments in parentheses indicate significant differences between different treatment conditions. As(V): sodium arsenate dibasic heptahydrate; As(III): sodium arsenite; T: treatment; G: genotype; T × G: treatment by genotype interaction

To investigate the effect of *OsGSTU40* on As accumulation in different plant tissues, we measured the As concentrations in both roots and shoots. However, under control conditions, As concentrations were below the detection limits (Fig. 6 A–D).

Under acute stress, higher accumulations of As in both shoots and roots were observed in the As(V) treatment than in the As(III) treatment. At the tissue level, higher amounts of As were found in the roots than in the shoots of all genotypes (Fig. 6 A and C). Interestingly, in the As(V) treatment, OE lines showed significantly higher concentrations of shoot As (167 ± 12 mg kg^−1^) than WT (92 ± 38 mg kg^−1^) (Fig. 6 A). In roots, both OE lines showed lower As concentrations in the As(III) treatment than the WT. However, a significant difference was found only in OE #2 compared to WT.

Under chronic stress, As(III) treatment led to higher As accumulation in both roots and shoots than As(V) (Fig. 6 B and D). Genotypic differences were observed for shoot As concentrations in the As(V) treatment. WT showed approximately four times lower As concentrations than OE lines (Fig. 6 B). However, no significant differences in As concentration in roots were found between WT and OE lines in both As(III) and As(V) treatments (Fig. 6 D).

Irrespective of the As concentration and the species used, root tissues accumulated more As than shoot tissues. Overall, the results of As analyses within plant tissues revealed that As(III) at higher concentrations was detrimental to plants, whereas the same doses of As(V) took a longer time to induce stress symptoms in rice plants (Fig. 2 E). Even with the presence of lower doses of As in rice growing nutrient solution, long-term exposure led to more accumulation of As inside the plant (Fig. 6 B and D).

### As stress affects the uptake of essential elements

**Fig. 7:**
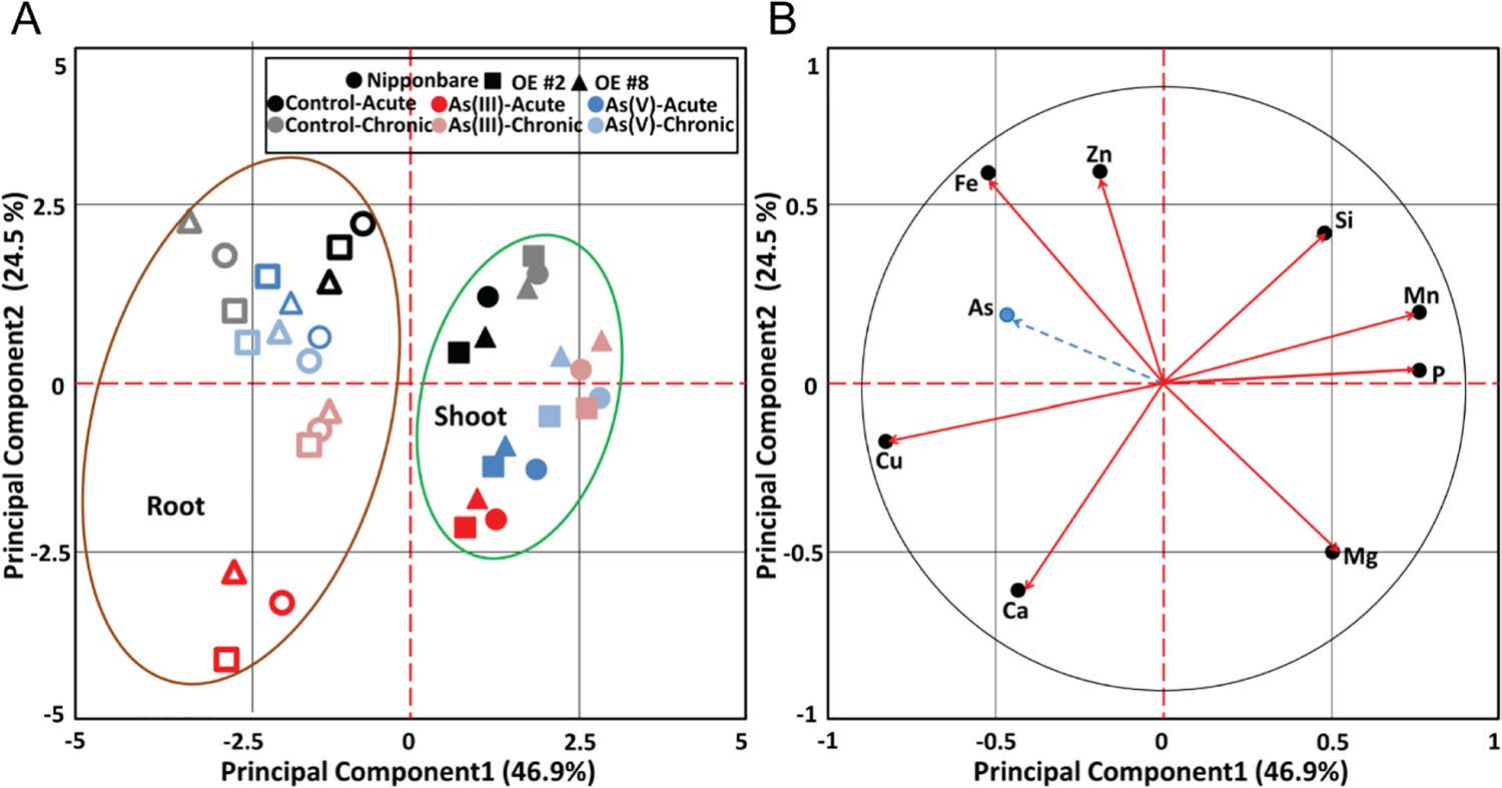
Principal component analysis for element uptake under arsenic stress conditions. **A** Score plot showing principal component analysis (PCA) for the first two principal component (PC) scores, PC1 vs. PC2 describing the total element uptake in roots and shoots measured for all genotypes under acute and chronic arsenic stress. **B** Loading plot showing the vector coefficients of element concentration variables for the first principal component vs. the coefficient for the second principal component

PCA was performed to identify how As treatments in rice plants affect the mineral composition of plants, including essential nutrients such as calcium (Ca), copper (Cu), iron (Fe), manganese (Mn), magnesium (Mg), phosphorus (P), silicon (Si), and zinc (Zn). PCA explained 71.4% of the total variation (Fig. 7) in element concentrations in different tissues (shoot and root) of various genotypes under distinct As treatments. Principal component 1 (PC1) accounted for 46.9%, and principal component 2 (PC2) accounted for 24.5%. Shoot and root tissues were clearly distinguished in terms of their element composition in different treatments. PC2 separated the different arsenic treatments, which were dominant over the genotypes in terms of element composition (Fig. 7 A). Figure 7 B represents the relations of As to other elements. Arsenic was positively correlated with Fe and Zn, which indicates that increased uptake of As appears to facilitate increased uptake of Fe and Zn. In contrast, As uptake was negatively correlated with the uptake of P, Mn, Si, and Mg. Higher uptake of As causes reduced uptake of elements such as P and Si.

## Discussion

In this study, we evaluated the toxic effects of As(III) and As(V) on rice plants differing in *OsGSTU40* transcription at the phenotypic, biochemical, and molecular levels. Two stress types, acute (10 mg L^−1^ As for ten days) and chronic (2 mg L^−1^ As for 20 days), were established. These stress intensities correspond to As concentrations commonly prevailing in the pore water of affected rice fields (Abedin et al. 2002; Cao et al. 2017; Murugaiyan et al. 2021, 2019; Yu et al. 2012; Zhang et al. 2008). Screening experiments were conducted in hydroponic systems without aeration to simulate anaerobic conditions in paddy fields. Therefore, our results are relevant to typical rice-growing conditions.

The first aim of this study was to differentiate rice plant responses to As(III) and As(V) at different concentrations. Our study clearly showed that distinct As species affect rice plants differently, depending on their concentrations. Under acute stress, we found that As(V) treatment led to significantly higher As accumulation in both shoot and root tissues than As(III), while the opposite was observed under chronic stresses (Fig. 6). Plants take up As(V) through phosphate transporters (Catarecha et al. 2007; González et al. 2005; Zhao et al. 2010), while As(III) is taken up via aquaglyceroporin channels, e.g., OsNIP1;1, OsNIP3;1, OsNIP3;2, silicon transporter Lsi1(OsNIP2;1), and Lsi2 (Ma et al. 2008). In this study, we used a hydroponic system to mimic anaerobic paddy field conditions, which can readily reduce As(V) to As(III). Part of the As(V) may have been reduced to As(III) during the 10-day treatment time slot, and the significantly higher As accumulation in plants grown in As(V) treatment might be caused by more efficient uptake of As mediated by both phosphate and silicon transporters. However, under chronic stress, the effects from the co-functions of both types of transporters might become less significant since As(III) is taken up as uncharged arsenous acid via the highly expressed silicon transporters, which have low selectivity but high transport rates (Ma et al. 2008; Meharg and Jardine 2003). We should also consider the possible ‘concentration’ effect due to the markedly lower shoot and root biomass (Fig. 2 and Table 1) in the As(III) chronic treatments.

Despite the lower shoot As accumulation in the As(III) acute treatment, the toxic effects of As(III) on rice plants regarding the reduction of shoot and root growth (Fig. 2 and Table 1) and shoot MDA concentration (Fig. 5 A) were more significant than those of As(V). Similar results were reported in a study conducted under field conditions (Kumar et al. 2022). In addition to the negative effects on plants at the vegetative growth stage, a previous study by Abedin and Meharg (2002) reported that As(III) showed higher toxic effects on rice seed germination than As(V). Under chronic stress, As(III) treatment affected morphological traits more severely than As(V) (Fig. 2 B and D and Table 1). Altogether, our results agreed with other previous studies (Abedin et al. 2002; Chakrabarty et al. 2009) that As(III) treatment causes more toxic effects than As(V) on rice plants.

The toxic effects caused by As(V) were associated with interference with plant phosphate metabolism, e.g., ATP synthesis and protein phosphorylation (Hughes 2002). As(III) binds to thiol groups in proteins, enzymes, and other biomolecules, such as glutathione, which is an important antioxidant in plant cells, leading to oxidative stress (Ma et al. 2008). In addition, As(III) can disrupt the electron transport chain in photosynthesis and respiration, causing cell death (Farooq et al. 2016). However, analysis of As speciation found that the majority of As (> 90%) in plant tissues is As(III) because As(V) is rapidly reduced to As(III) in roots (Carey et al. 2010). Therefore, the toxic effects of As(V) in plants may be due to its reduced product As(III) (Zhao et al. 2010). More studies are warranted to truly understand the differences in toxic effects between As(III) and As(V).

The second major aim of this study was to characterize the role of *OsGSTU40* in plant responses to different types of As stress. Plant GSTs are capable of catalyzing the conjugation of the GSH moiety on the hydrophobic and electrophilic centers of xenobiotic compounds, including As (Labrou et al. 2015). Previous analyses have found that As treatment highly induced the transcript level of a number of *GST* genes, including *OsGSTU40* (Chakrabarty et al. 2009; Norton et al. 2008)*,* which was confirmed by our study. *OsGSTU40* belongs to the tau-class, the largest subfamily of plant GSTs (Fig. 1) that have been reported to be involved in the detoxification of various heavy metals and metalloids (Chakrabarty et al. 2009; Matthus et al. 2015; Srivastava et al. 2019; Tiwari et al. 2022; Tripathi et al. 2014). We hypothesized that overexpression of *OsGSTU40* mitigated As toxicity, which was evaluated by plant development and MDA formation. Our results suggested that overexpressing *OsGSTU40* in two OE lines led to better plant growth in both As(V) and As(III) treatment (Fig. 2 A and C and Table 1) and enhanced oxidative stress tolerance to As(III) under acute stress (Fig. 5 A). A recent study by Tiwari et al. (2022) reported that another member of the rice GST tau-class, *GSTU5*, conferred tolerance to As(V) in *E. coli*, rice calli, and seedlings. As(V) treatment significantly induced crude GST activity, which agreed with our findings in both acute and chronic As(V) treatment (Fig. 4). In addition, we investigated the responses of GST activity to As(III), which turned out to be dependent on the stress intensity and duration, i.e., suppressed by acute stress but induced by chronic stress (Fig. 4). The decreased GST activity in acute As(III) treatment might be caused by the depletion of intracellular glutathione to conjugate As(III) (Clemens and Ma 2016; Zhao et al. 2010). The elevated transcription level of *OsGSTU40* in OE lines (ca. 250-times higher than WT) conferred tolerance, mainly observed in the acute stresses (Fig. 2 A and B). In particular, overexpressing *OsGSTU40* mitigated the oxidative stress (Fig. 5 A) caused by As(III) despite similar shoot As accumulation between the WT and OE lines (Fig. 6 A). Likewise, OE lines showed similar MDA concentrations under both acute and chronic stresses of As(V) (Fig. 5), although shoot As concentrations were significantly higher compared to WT (Fig. 6 A and B). The increased As accumulation in OE lines is likely a result of vacuolar sequestration of the conjugated As-GSH or other As-PC compounds mediated by *OsGSTU40*. Altogether, the tolerance mechanisms associated with *OsGSTU40* were shoot-based, i.e., OE plants exhibited less oxidative stress despite shoot As accumulation similar to or even higher than that in WT plants. Therefore, we identified a distinct shoot-based tolerance mechanism that differs from the study by Tiwari et al. (2022), where *OsGSTU5* OE lines were found to store excess As in the roots to limit transport into shoot tissues.

Overall, the results suggested that in the absence of As, both WT and OE lines had similar growth patterns, but in the presence of As, *OsGSTU40* OE lines performed better than WT. The mitigating effect of *OsGSTU40* overexpression in terms of oxidative stress and plant growth was particularly pronounced when plants were exposed to the more toxic As(III), which is the predominant form that occurs in flooded rice production. Our data also demonstrated that As(III) is substantially more toxic to rice plants than As(V). Although these results are promising, further investigations will be required to employ *OsGSTU40* as a target for As tolerance breeding. In the case of As(V), *OsGSTU40* overexpression was associated with elevated As uptake into shoots, which is undesirable because As should be excluded from entering the food (or feed) chain. In particular, the translocation of elevated levels of As into grains needs to be excluded. Therefore, *OsGSTU40* overexpression may be a more suitable strategy for flooded rice production systems where As(III) is dominant. This approach should be combined with other mechanisms of As exclusion to eventually develop arsenic-safe rice.

## Supplementary Information

Below is the link to the electronic supplementary material.

Supplementary file1 (PDF 658 KB)

## Data Availability

Data that support the findings and plant materials in this study are available from the corresponding author upon reasonable request.
