# Supplementary material for: Differential effects of arsenite and arsenate on rice (Oryza sativa) plants differing in glutathione S-transferase gene expression
Source: Environ Sci Pollut Res Int. 2023 Jul 24;30(40):92268–81. doi: 10.1007/s11356-023-28833-x (PMC10447600; doi:10.1007/s11356-023-28833-x)
Supplement: Supplementary file 1 — Supplementary file1 (PDF 658 KB) [file 11356_2023_28833_MOESM1_ESM.pdf]

Supplementary Data

Table S1: Full strength nutrient solution used for hydroponics experiment.

**Element**	**Chemical**	**Chemical Formula**	**Concentration of element in final nutrient solution (ppm)**
N	Ammonium nitrate	NH_4NO_3	40
P	Sodium dihydrogen phosphate dihydrate	$NaH_2O_4 \cdot 2H_2O$	10
K	Potassium sulphate	K_2SO_4	40
Ca	Calcium Chloride	$CaCl_2$	40
Mg	Magnesium sulfate heptahydrate	$MgSO_4 \cdot 7H_2O$	39.94
Mn	Manganese Chloride Tetrahydrate	$MnCl_2 \cdot 4H_2O$	0.52
Mo	Ammonium heptamolybdate tetrahydrate	$(NH_4)_6\ Mo_7O_{24} \cdot 4H_2O$	0.05
B	Boric Acid	H_3BO_3	0.2
Cu	Copper sulfate pentahydrate	$CuSO_4 \cdot 5H_2O$	0.01
Fe	Ferric Sodium EDTA	Fe-Na-EDTA	1.98
Zn	Zinc sulfate heptahydrate	$ZnSO_4 \cdot 7H_2O$	0.01
Si	Potassium Silicate	K_2SiO_3	1.40

Table S2: Primers used in this study.

**Oligo name**	**Forward (5'-3')**	**Reverse (5’-3')**
Genotyping primers		
Bialaphos resistance gene (*bar*)	GAAGTCCAGCTGCCAGAAAC	AAGCACGGTCAACTTCCGTA
Quantitative real-time PCR		
Ubiquitin-40s ribosomal protein (*UBQ5*)	ACCACTTCGACCGCCACTACT	ACGCCTAAGCCTGCTGGTT
Glutathione S-Transferease (*LOC_Os01g49710*) transcription	CCTACTTCGACAGCACGTTC	CTTCCCCTTGGAGCACTC

Supplementary Figure

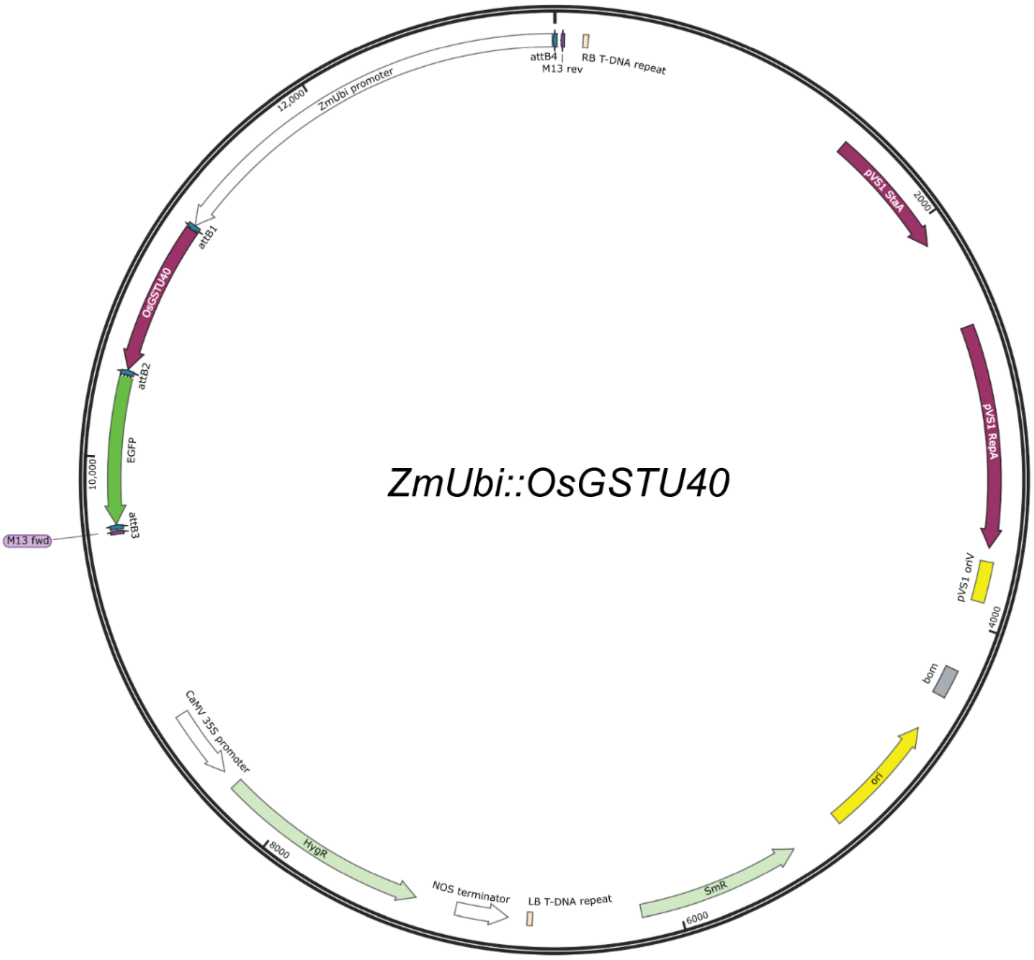

Figure S1. Map of *OsGSTU40* overexpression vector. Coding sequence of *OsGSTU40* was synthesized and cloned into a Gateway expression vector using LR clonase. *OsGSTU40* transcription was driven by a strong promoter of *ZmUbi* (maize ubiquitin).

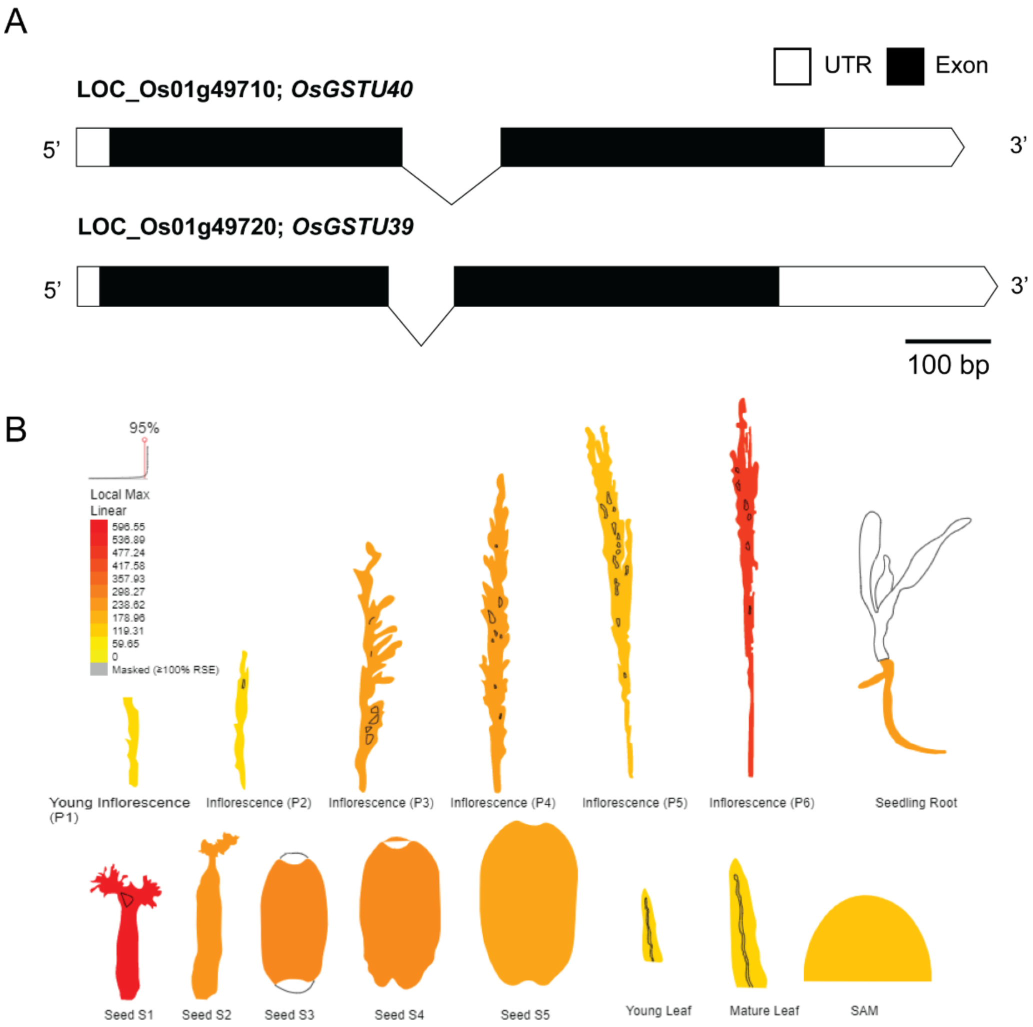

Figure S2. Gene structure (A) of *LOC_Os01g49710* (*OsGSTU40*) and *LOC_Os01g49720* (*OsGSTU39*). (B) *OsGSTU40* mRNA abundance in rice tissues. Data was obtained from the ePlant database (http://bar.utoronto.ca/eplant_rice/).

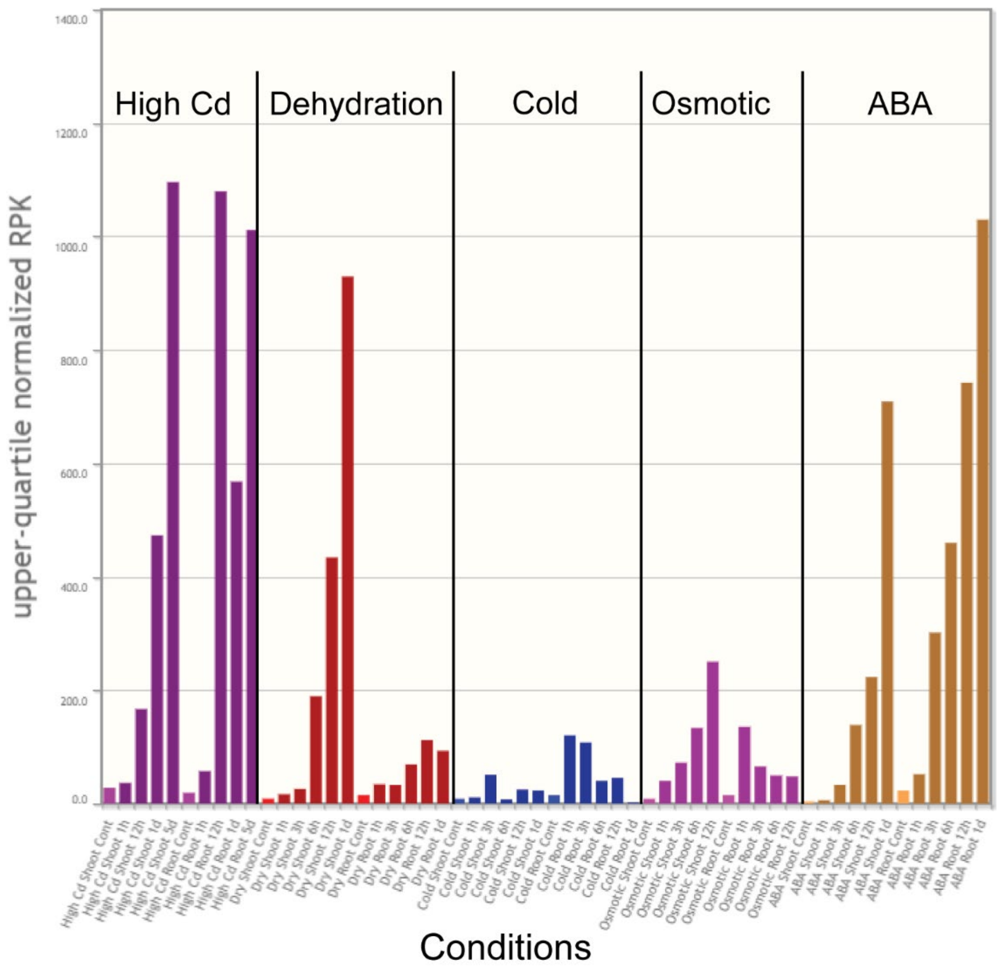

Figure S3. Responses of OsGSTU40 transcript level to different abiotic stresses and abscisic acid treatment. Data was obtained from the TENOR database (https://tenor.dna.affrc.go.jp/EPV/Os01t0692000-01/).

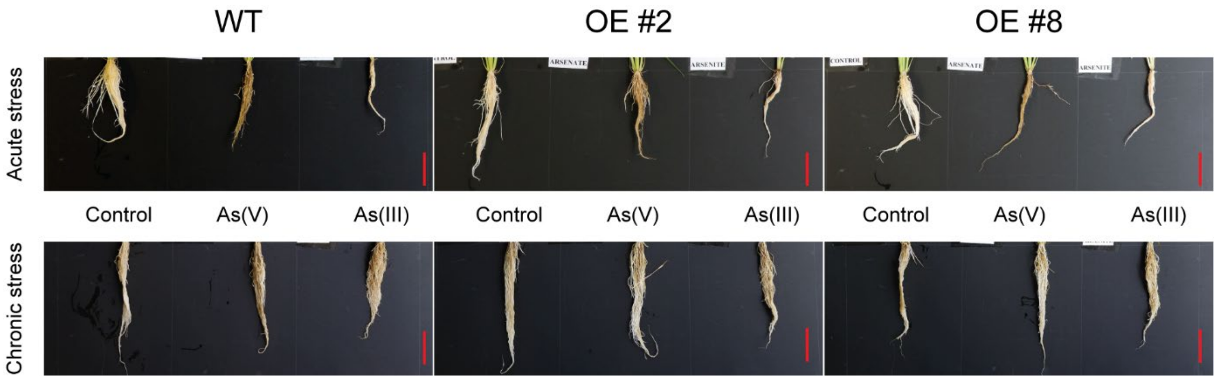

Figure S4. Phenotype of WT and OE lines in acute and chronic arsenic stress.
